# Biomechanical evaluation of oversized drilling technique on primary implant stability measured by insertion torque and resonance frequency analysis

**DOI:** 10.4317/jced.52873

**Published:** 2016-07-01

**Authors:** Gorka Santamaría-Arrieta, Aritza Brizuela-Velasco, Felipe J. Fernández-González, David Chávarri-Prado, Yelko Chento-Valiente, Eneko Solaberrieta, Markel Diéguez-Pereira, José-Antonio Vega, Jaime Yurrebaso-Asúa

**Affiliations:** 1 Professor Department of Stomatology I, School of Medicine and Dentistry, University of the Basque Country, Leioa, Spain; 2 Professor Department of Surgery and medical-surgical specialties, University of Oviedo, Oviedo, Spain; 3 Professor Department of Orthodontics and Dentofacial Orthopedics, University of Oviedo, Oviedo, Spain; 4 Graduate student. Engineering Physics Department of Nuclear Engineering and Fluid Mechanics, Engineering School, University of the Basque Country, Bilbao, Spain; 5 Researcher. Department of Graphic Design and Engineering Projects, University of the Basque Country UPV/EHU, Bilbao, Spain; 6 Student of oral implantology degree in the University of the Basque Country, Leioa, Spain; 7 Professor and full chair. Department of morphology and Cell Biology, University of Oviedo, Spain; 8 Professor. Facultad de Ciencias de la Salud, Universidad Autónoma de Chile, Chile

## Abstract

**Background:**

This study evaluated the influence of implant site preparation depth on primary stability measured by insertion torque and resonance frequency analysis (RFA).

**Material and Methods:**

Thirty-two implant sites were prepared in eight veal rib blocks. Sixteen sites were prepared using the conventional drilling sequence recommended by the manufacturer to a working depth of 10mm. The remaining 16 sites were prepared using an oversize drilling technique (overpreparation) to a working depth of 12mm. Bone density was determined using cone beam computerized tomography (CBCT). The implants were placed and primary stability was measured by two methods: insertion torque (Ncm), and RFA (implant stability quotient [ISQ]).

**Results:**

The highest torque values were achieved by the conventional drilling technique (10mm). The ANOVA test confirmed that there was a significant correlation between torque and drilling depth (*p*<0.05). However, no statistically significant differences were obtained between ISQ values at 10 or 12 mm drilling depths (*p*>0.05) at either measurement direction (cortical and medullar). No statistical relation between torque and ISQ values was identified, or between bone density and primary stability (*p* >0.05).

**Conclusions:**

Vertical overpreparation of the implant bed will obtain lower insertion torque values, but does not produce statistically significant differences in ISQ values.

** Key words:**Implant stability quotient, overdrilling, primary stability, resonance frequency analysis, torque.

## Introduction

Several different techniques are used for implant bed preparation. The standard procedure consists of an increasing drilling diameter sequence, performed with a micro-motor, in order to create a bed into which the implant will be inserted (1). Each implant manufacturer recommends a specific drilling system with a drilling sequence. These conventional drilling techniques are effective, reliable and relatively simple to perform. In the conventional approach to preparing implant sites, depth and length of the bed corresponds to the implants’ dimensions. But this is not always the case. When flapless surgery is performed in the aesthetic zone, with abundant available bone, some clinicians prefer to perform an oversize drilling technique in apical direction so that they may choose between crestal or subcrestal implant placement depending on the soft tissue situation. Nonetheless, if crestal placement is chosen, oversize drilling to create additional depth may influence the implant’s primary stability. Several studies have discussed the influence of preparation/bed width on insertion torque or implant primary stability (2-4). But scientific evidence on the influence of implant bed depth remains scarce (5,6).

Primary stability, defined as the absence of clinical mobility, has been widely acknowledged as a key factor in achieving and maintaining osseointegration (7); this is influenced by bone density, implant shape, and the surgical technique employed (8). Primary stability can be measured by different methods (4): biomechanical tests, including insertion and disinsertion torque measurements, and non-invasive techniques such as resonance frequency analysis (RFA). RFA makes it possible to measure implant stability without damaging the bone-implant junction. It is based on the application of controlled bending loads, through a small transducer attached to the implant or implant abutment, which imitates a functional load and its direction, and provides information about the flexibility of the bone-to-implant union. In this way, a series of measurements during the osseointegration period should monitor increasing implant stability as a consequence of the remodeling process (5,9). With the first-generation RFA machines, results were expressed in hertz but more recently data are converted into implant stability quotient (ISQ) units. ISQ values vary between 1-100, 1 being the lowest value and 100 the highest (8).

Many authors affirm that there is a correlation between insertion torque and ISQ values (4,10-12). Nevertheless, this correlation does not always occur as sometimes, high torque values do not correspond to high ISQ values, and likewise, low torque values do not always correspond to low ISQ values (13-16).

The objectives of this *in vitro* experimental study were to determine the influence of implant site preparation depth on primary stability measured by ISQ values, and to determine whether there is a correlation between implant primary stability measured by peak insertion torque and by ISQ values.

## Material and Methods

-Specimens: Eight veal ribs were selected from an animal sacrificed at the age of 14 months and weighing 276 kg, intended for human consumption. This bone model is comparable to an edentulous human jaw due to its macroscopic composition of medullar and cortical bone (17). The middle section of the ribs has been shown to more closely resemble type 1-2 intermediate quality bone, yielding a suitable model for human edentulous bone (18). The day after animal sacrifice, the ribs were collected and all soft tissue removed. Then, the central parts were cut into 60 mm length blocks with a power micro-saw, and the ends were removed. Once the blocks were ready, they were conserved following the protocol described by Tricio *et al.* (19). Bone blocks were submerged in 50% ethanol and saline solution and kept at room temperature for five days. Storage in this solution prevents decreases in the bone’s Young´s modulus greater than 2% for up to nine months. Fifteen hours before initiating the experiment procedure, the bone blocks were submerged in saline solution for 12 hours. They were then covered with saline soaked gauze for three hours. Finally, the upper cortical bone was removed by a hand-piece and bur with abundant irrigation to obtain type III-IV bone according to Lekholm & Zarb´s classification (18). The research protocol was reviewed and approved by the Ethical Committee of the Hospital of León, Spain (#1586).

**Figure 1:**
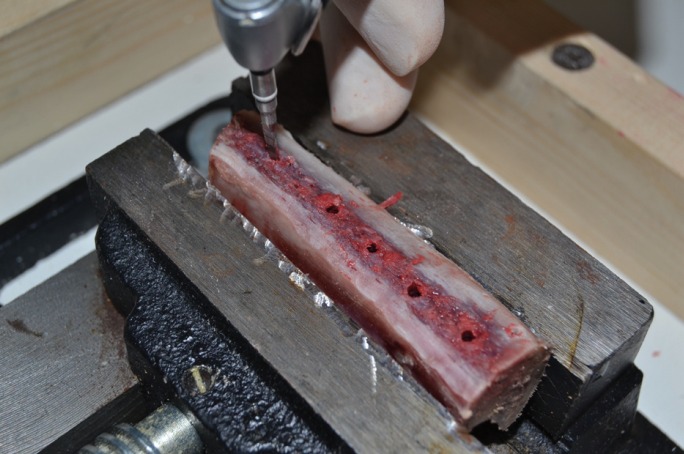
Surgical procedure: locating implant positions with the cortical drill.

-Study procedure: Bone blocks were fixed in a custom made device and implant site preparation was performed as follows: first, a lanceolate drill was used to mark the positions of the implants and then with a 1.8 mm start-up drill, beds were prepared to one of the two different working depths: 10mm (Group A) and 12mm (Group B). Bed preparation continued following the drilling sequence recommended by the manufacturer, ending with a 3.6 mm drill (Fig. 1).

Then, the specimens were scanned using a cone beam computerized tomography (CBCT) scanner at 105 kV and 8mA, with a 120 mm x 80 mm field of view (FOV) (Carestream 8100 3D, Carestream Health, NY, USA). The images were processed with two different imaging programs for bone density assessments in Hounsfield Units (HU): i•Dixel (J. Morita, Kyoto, Japan) and Carestream Dental Imaging Software (Carestream Health. NY, USA). HU were measured in a 1 mm wide circular corridor 4 mm around the central long axis of the preparation site, as described in the literature (20).

**Figure 2:**
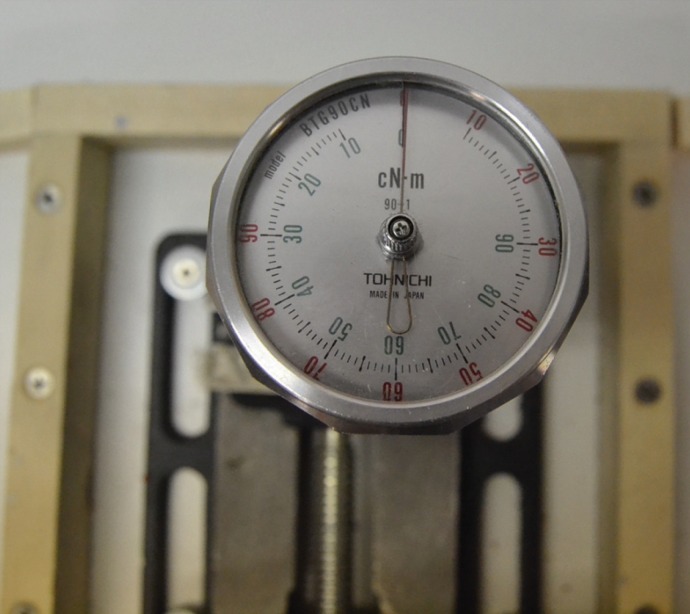
Implant primary stability measured by peak insertion torque with a manual torque wrench.

**Figure 3:**
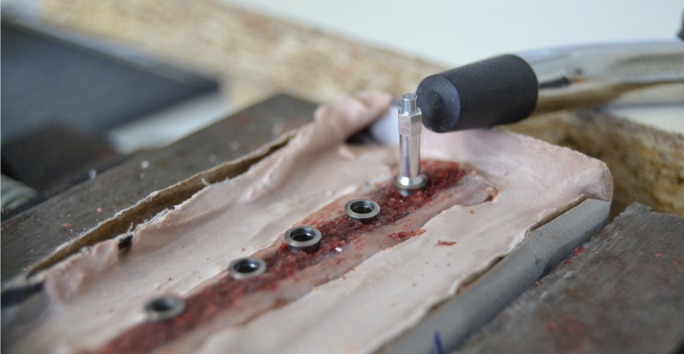
Implant primary stability measured with resonance frequency analysis (Osstell ISQ).

**Figure 4:**
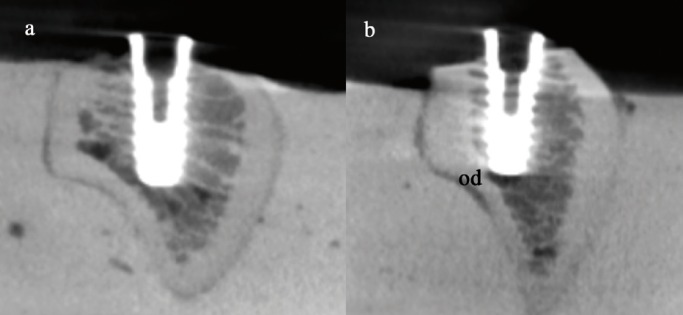
a) CBCT images of a Group A implant and b) An implant of group B. Od: Over-drill.

The bone blocks were then embedded in plaster and fixed in a custom made rig, designed to avoid the pressure of a clamp that might skew RFA measurement. Four mm diameter and 10 mm length Klockner Essential Cone implants (Soadco, Escaldes-Engordany, Andorra) were inserted in each prepared site with each polished collar in supra-crestal position. Peak insertion torque was measured with a previously calibrated manual torque wrench (model BTG90CN, Tohnichi. Tokyo, Japan) (Fig. 2) and RFA was measured with an Osstell third generation instrument (Osstell, Gothenburg, Sweden) (Fig. 3) perpendicular and parallel to the long axis of the bone blocks (cortical and medullar respectively). Finally, the specimens were scanned again with a CBCT in order to check that the implants were not anchored in cortical bone (Fig. 4).

-Statistical analysis: Descriptive statistical analysis was performed, obtaining central trending and dispersion values. Analysis of variance (ANOVA) was carried out to evaluate the differences between the two drilling techniques. Pearson’s Correlation Coefficient was used to determine the relation between quantitative variables (insertion torque and ISQ values). Statistical significance was set at *p*<0.05.

## Results

The distribution of torque, ISQ, and bone density values obtained is summarized in  Table 1.

**Table 1:**
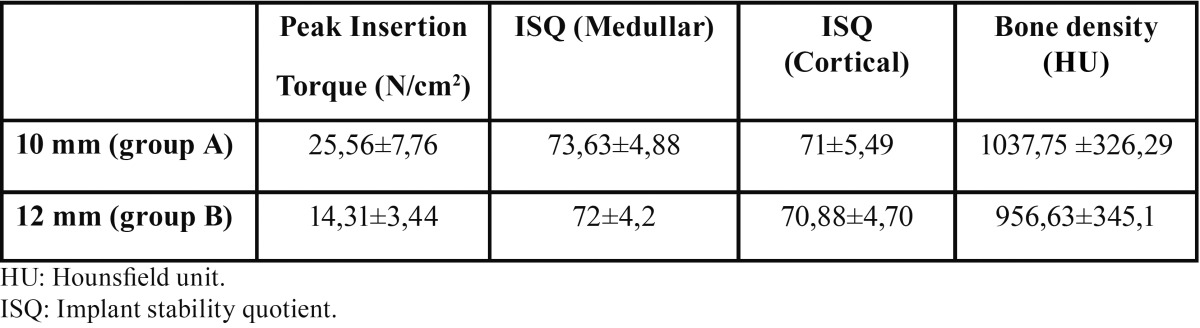
Peak insertion torque, ISQ values, bone density mean values and standard deviation of groups A and B.

The highest torque values were achieved by implants inserted in the conventionally drilled sites (Group A: 25.56±7.76 N/cm2 / Group B: 14.31±3.44 N/cm2). ISQ values were higher for Group A implants (73.63±4.88 medullar / 71±5.49 cortical) than Group B implants (72±4.20 medullar/70.88±4.70 cortical).

While the ANOVA test showed a significant positive correlation between peak insertion torque and site preparation depth (*p*<0.05), no statistically significant relation was found between ISQ and preparation depth (*p*>0.05). Furthermore, no significant statistical relation was found between peak insertion torque and ISQ values (*p*>0.05). Regarding the influence of bone density on primary stability, the results suggest that there was no statistically significant relation between HU and peak insertion torque or ISQ (*p*>0.05). Mean bone density values were 1037.75±326.29 HU for Group A and 956.63±345.1 HU for Group B.

## Discussion

The term primary stability refers to micromovement. When dental implants exceed a certain threshold of micromotion, a predominance of fibrous encapsulation can be expected at the expense of osseointegration. According to a literature review published by Szmukler-Monkler *et al.* (1998), this threshold is located between 50 and 150 μm movement (21). Any mobility over this limit induces the formation of a fibrous membrane around the implant, which inhibits its osseointegration and favors implant failure (22).

This statement is based on several articles published by different authors: Soballe *et al.* and Pilliar *et al.* showed that movements of over 500 μm and 150 μm respectively could be regarded as excessive, and so harmful to bone growth around implants (23,24).

Pilliar *et al.* have recently discovered that micro-movement of up to 50 μm is well tolerated by the supporting bone tissue; this finding represents a higher tolerance limit than previously believed. Therefore, the micromovement tolerated by an implant without repercussions for osseointegration, is somewhere between 50 and 150 μm, probably around 100 μm, as proposed by Brunski *et al.* (25).

Given the significance of implant micromovement, it is clear that achieving maximum primary stability at the moment of insertion is of prime importance. So, in addition to bone density and implant design, it is important to determine which surgical technique will provide maximum primary stability.

In the present study, preparation depth prior to implant insertion showed contradictory results regarding the primary stability obtained. Although wide differences were observed in torque insertion values between the two drilling depths analyzed, significant differences in ISQ values were not observed. This could be explained by the fact that in absence of an apical stop, the mechanical resistance that the preparation walls exert on the implant during its insertion decreases. But ISQ values were not affected, although the preparation depth varied given that RFA measures the degree of lateral displacement of the implant into the bone (20). These results are in contrast to another study performed in cow ribs, in which three different bone preparation techniques were compared: preparation depth equal to implant length, 1 mm more than implant length, and 1 mm less; the study also compared cylindrical and conical implants. For cylindrical implants, significant differences resulting from the different drilling depths were not found. But for conical implants, the highest ISQ values were observed in the 1 mm less than implant length samples, followed by the standard preparations, and the over-drilled preparations. These differences were similar for bone types II and IV, although the primary stability values achieved were lower in all groups for type IV bone (5).

The second objective of this study was to establish the relationship between two measurement methods: insertion torque and RFA. The results showed an absence of statistical correlation between insertion toque and RFA data which is in accordance with Several authors that have obtained similar results (14,16,26-28). While others have concluded that there is a correlation between insertion torque and RFA (29,30). The reason for these different conclusions could be that insertion torque and RFA data are not the result of the same biomechanical circumstances – they do not measure the same kind of stability. Insertion torque could be defined as the resistance that an implant undergoes to rotational advance in apical direction around its axis. RFA is a non-invasive method that makes objective measurements of the stiffness of the bone-implant union and of the implant’s lateral displacement when a load is applied (8,20). From the biomechanical point of view, it could be expected that the relationship between insertion torque or RFA with bone density would be predictable. Furthermore, it could be expected that insertion torque is related to implant macro-design and modifications to surgical technique, and RFA to bone availability and the presence of coronal cortical bone. Regarding the latter, CBCT performed before implant insertion, observed an homogeneous morphology between all the ribs: the medullar bone was always surrounded by thick cortical bone, especially in its coronal portion (a mean of 2.63 mm).

The limitations of the present study design are that the bone specimen conservation technique does not guarantee complete conservation of the bone’s mechanical properties, and for obvious reasons there are other factors such as vascularization or the presence of soft tissues that could be relevant but which have not been tested. Nevertheless, the study design did not differ greatly from other similar experimental researches (5,6,16-20). But clearly, the present findings need validation by means of clinical research of adequate design.

In spite of the study’s limitations, it may be concluded that when vertical over-preparation of the implant bed is performed, lower insertion torque values are obtained, but oversize drilling or conventional drilling depths do not produce statistically significant differences in ISQ values. Therefore, although mechanical resistance to implant insertion in over-prepared bone decreased, primary stability was not affected. Furthermore, no relation was observed between implant primary stability measured by peak insertion torque and ISQ. *In vivo* studies are required to understand the actual clinical situation in which many biological factors influence the primary stability of implants.
